# COPD-associated proteins as biomarkers and drug targets: Insights from genetic and proteomic analyses

**DOI:** 10.1097/MD.0000000000045102

**Published:** 2025-10-10

**Authors:** Xuejiao Lin, Qingxiu Zheng, Jiangpo Ma, Wenqi Ouyang, Binbin Chen

**Affiliations:** a Department of Medical Respiratory, Wenzhou TCM Hospital of Zhejiang Chinese Medical University, Wenzhou, China; b Cixi Biomedical Research Institute, Wenzhou Medical University, Zhejiang, China; c Department of General Practice, The Second People’s Hosptial of Yichang, China Three Gorges University, Yichang, China.

**Keywords:** biomarkers, cis-pQTL, COPD, lifestyle factors, Mendelian randomization, protein–protein interaction, therapeutic targets

## Abstract

Chronic obstructive pulmonary disease (COPD) is a major global health burden with significant morbidity and mortality. Identifying circulating protein biomarkers may help understand COPD pathogenesis and develop novel therapeutic targets. We conducted a 2-sample Mendelian randomization (MR) analysis to assess the causal relationship between circulating proteins (cis-pQTLs) and COPD. Co-localization analysis confirmed shared genetic variants, while tissue-specific and pathway analyses provided insights into the biological roles of key proteins. Protein–protein interaction network analysis was conducted, and the impact of lifestyle factors on COPD-related proteins was also evaluated. Mendelian randomization analysis identified 18 proteins in the Decode dataset and 8 in the UK Biobank Pharmaceutical Proteomics Project dataset that were significantly associated with COPD after false discovery rate correction, with 4 proteins overlapping across datasets. Co-localization analysis provided strong evidence for proteins such as MMP12, KLC1, and apolipoprotein E being causally linked to COPD. Pathway analyses highlighted the involvement of these proteins in respiratory and inflammatory processes. Lifestyle factors, including dietary habits, were found to influence COPD-related proteins, suggesting avenues for lifestyle-based intervention. This study identified multiple circulating proteins associated with COPD, highlighting their potential as biomarkers and therapeutic targets. The findings suggest that targeted lifestyle interventions could modulate key proteins involved in COPD pathogenesis, providing opportunities for personalized management and prevention strategies.

## 1. Introduction

Chronic obstructive pulmonary disease (COPD) is a major global health burden, characterized by persistent respiratory symptoms and irreversible airflow limitation.^[1]^ It is estimated that COPD affects over 300 million people worldwide, contributing significantly to morbidity, mortality, and healthcare costs.^[2]^ COPD is a leading cause of disability and the third leading cause of death globally, posing a significant challenge to public health.^[3]^ Despite the substantial impact of COPD, the underlying molecular mechanisms driving its pathogenesis remain incompletely understood, hindering the development of effective targeted therapies.^[4]^

Cigarette smoking is the primary risk factor for COPD, accounting for up to 90% of cases.^[5]^ However, only a subset of smokers develops clinically significant COPD, suggesting the involvement of complex genetic and environmental factors in disease susceptibility and progression.^[6]^ In recent years, emerging evidence has highlighted the potential role of circulating proteins as important biomarkers and pathogenic mediators in COPD.^[7]^ Genetic variations that influence protein levels, known as protein quantitative trait loci (pQTLs), offer a unique opportunity to explore the causal relationships between specific proteins and COPD susceptibility through Mendelian randomization (MR) analysis.^[8]^

MR is a powerful epidemiological approach that leverages genetic variants as instrumental variables to assess the causal effect of an exposure (in this case, circulating protein levels) on an outcome (COPD risk).^[9]^ By utilizing pQTL data, researchers can assess the potential causal effect of proteins on disease risk, providing valuable insights into disease pathways and identifying novel therapeutic targets.^[10]^ This approach is particularly advantageous as it can help overcome the limitations of traditional observational studies, which are prone to confounding and reverse causation biases.^[11]^

This study aimed to comprehensively investigate the causal association between circulating proteins and COPD using a 2-sample MR approach.^[12]^ By integrating data from large-scale genetic studies, including the Decode and UK Biobank Pharmaceutical Proteomics Project (UKB-PPP) cohorts, the researchers sought to uncover robust protein biomarkers linked to COPD.^[13]^ These cohorts provided a wealth of genetic and proteomic data, enabling the systematic evaluation of thousands of circulating proteins and their potential causal roles in COPD.^[14]^

To strengthen the evidence for causality, the analysis also included co-localization analysis, which assessed whether the same genetic variants influence both protein levels and COPD susceptibility.^[15]^ This approach helped to identify shared causal variants, providing stronger support for the potential causal relationships between specific proteins and COPD.^[16]^

Furthermore, the study delved into the functional and biological contexts of the identified proteins through tissue-specific differential expression and pathway analyses.^[17]^ This comprehensive approach provided insights into the potential mechanisms by which these proteins may contribute to COPD pathogenesis, highlighting their relevance as therapeutic targets.^[6]^ By understanding the underlying biological pathways and tissue-specific expression patterns of these proteins, the researchers aimed to uncover novel insights into COPD pathology and identify promising therapeutic avenues.^[18]^

Importantly, the researchers also explored the impact of lifestyle factors on COPD-related proteins, seeking to identify proteins that could be modulated through lifestyle interventions. This aspect of the study offers promising avenues for developing personalized, lifestyle-based strategies to manage COPD risk and progression.^[19]^ By understanding the complex interplay between genetic factors, circulating proteins, and modifiable lifestyle behaviors, the researchers aimed to inform the development of more effective and targeted COPD management approaches. Overall, this comprehensive investigation of the causal relationship between circulating proteins and COPD, combined with the exploration of lifestyle influences, holds significant clinical and translational implications. The findings may inform the development of novel protein-based biomarkers, facilitate the identification of targetable pathways, and guide the design of lifestyle-focused interventions to improve COPD management and patient outcomes.

The identification of causal protein biomarkers could aid in the early detection of COPD, enabling earlier diagnosis and timely intervention.^[20]^ Furthermore, the elucidation of the functional roles of these proteins in COPD pathogenesis may uncover new therapeutic targets, paving the way for the development of targeted pharmacological treatments.^[21]^ Importantly, the insights gained from the exploration of lifestyle factors on COPD-related proteins could inform the design of personalized, non-pharmacological strategies, empowering patients to take an active role in managing their disease through lifestyle modifications.

In conclusion, this multifaceted study represents a significant advancement in the understanding of COPD pathogenesis, leveraging the power of genetic epidemiology and bioinformatics to identify causal protein biomarkers and explore their potential for clinical and therapeutic applications. By integrating diverse data sources and employing robust analytical approaches, the researchers have laid the groundwork for future studies aimed at translating these findings into improved COPD prevention, diagnosis, and management strategies, ultimately enhancing patient outcomes and reducing the global burden of this debilitating respiratory disease.

## 2. Materials and methods

### 2.1. Study design

This study is the first to investigate the association between circulating proteins (cis-acting protein quantitative trait loci [cis-pQTL]) and COPD using a 2-sample MR analysis. To strengthen the evidence for a causal relationship, we performed co-localization analysis to assess whether the same genetic variants influence both circulating protein levels and susceptibility to COPD. Additionally, we conducted tissue-specific differential expression and pathway analysis on the identified circulating proteins to further understand their biological roles in COPD.

**Figure 1.**
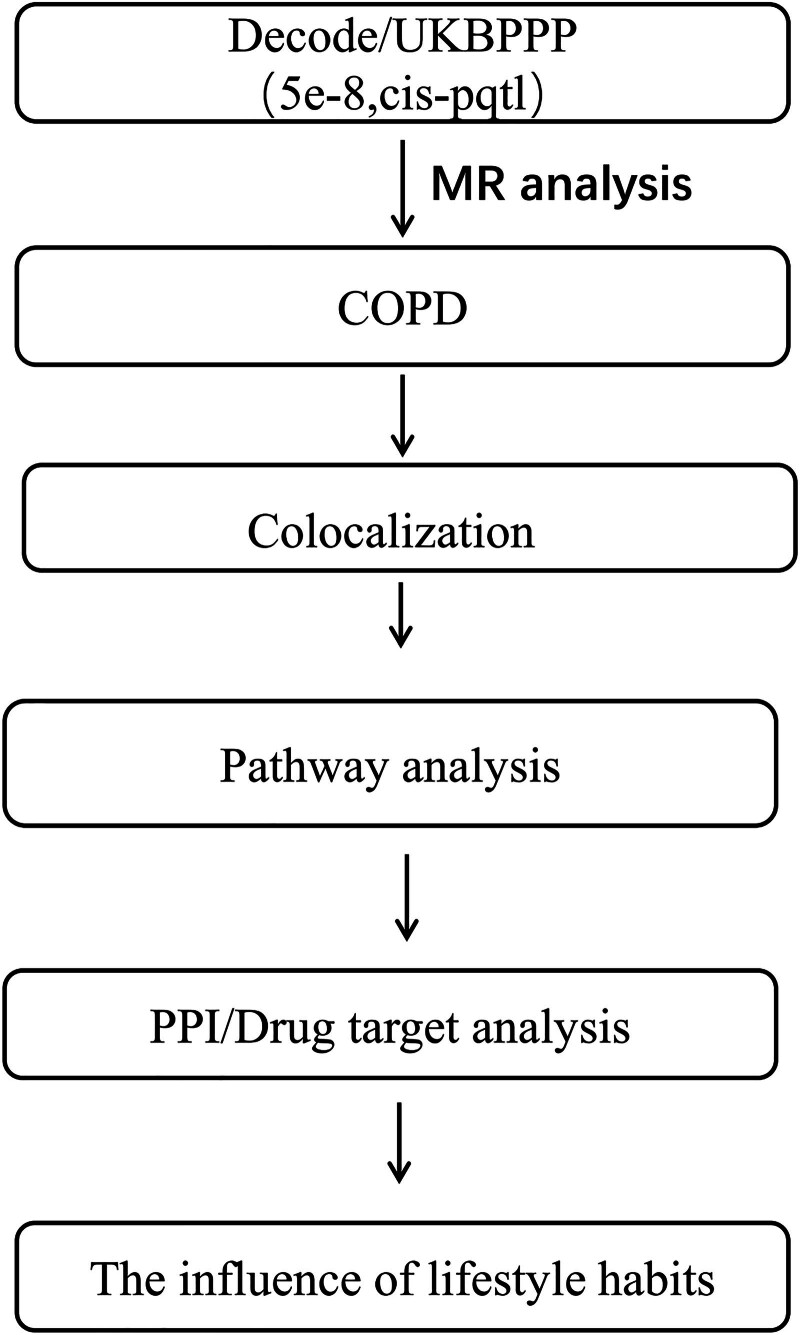
Flowchart illustrates the screening process for plasma proteins associated with COPD. The study utilizes a 2-sample MR analysis on data from Decode/UKB-PPP to investigate the causal relationship between circulating proteins and COPD. Co-localization analysis was performed to confirm shared genetic variants affecting protein levels and COPD susceptibility. This was followed by pathway and tissue-specific differential expression analyses to uncover the biological roles of identified proteins. A PPI and drug target analysis were conducted to prioritize proteins as potential therapeutic targets. Finally, a systematic MR approach examined the influence of lifestyle factors on COPD-related proteins, suggesting possible lifestyle-based intervention strategies. COPD = chronic obstructive pulmonary disease, MR = Mendelian randomization, PPI = protein–protein interaction, UKB-PPP = UK Biobank Pharmaceutical Proteomics Project

Following this, a protein–protein interaction (PPI) network analysis was performed to explore potential interactions among these proteins. This analysis was combined with drug efficacy evaluation to prioritize proteins that may serve as promising therapeutic targets for COPD. Finally, we applied a systematic MR approach to examine the relationship between healthy lifestyle factors and COPD-related proteins. This analysis aims to identify key proteins that could potentially be influenced by lifestyle modifications, thereby providing novel insights for lifestyle-based COPD intervention strategies (Fig. 1).

### 2.2. Data sources

We conducted a GWAS using data from 35,559 Icelandic individuals and 4907 aptamers. This analysis, based on the SomaScan platform, identified 28,191 genetic associations across 4907 aptamers.^[22]^ The participants came from 2 primary projects: the Icelandic Cancer Project, comprising 52% of the participants, and various genetic projects at Decode Genetics in Reykjavik, Iceland, accounting for the remaining 48%. Using recursive conditional analysis, we processed pre-calculated summary statistics to identify the most significant genetic variants in each region (±1 Mb), categorizing them as sentinel pQTLs (n = 18,084) and secondary pQTLs (n = 10,107).^[14]^

This GWAS achieved a high replication rate, corroborating 83% of reported pQTLs from the INTERVAL study (SomaScan-based) and 64% of pQTLs from the SCALLOP consortium (Olink-based). Given the extensive use of human proteins as drug targets, the integration of protein-level GWAS with disease-related GWAS data enables insights into genetic variations and their roles in disease mechanisms.^[23]^ Additionally, we leveraged data from the UKB-PPP Pharmaceutical Proteomics Project, a precompetitive biopharmaceutical consortium investigating plasma proteomic profiles of 54,219 participants from the UK Biobank. This initiative offers a comprehensive summary, including technical and biological validations, protein-disease associations, and predictive models for various demographic and health factors.^[24]^ The project has mapped quantitative trait loci (pQTLs) for 2923 proteins, uncovering 14,287 significant genetic associations, of which 81% were previously undocumented. This study highlights new ancestral-specific pQTLs, particularly among non-European individuals, and forecasts rapid pQTL discovery as sample sizes and mass spectrometry coverage expand.^[25]^

Furthermore, this project provides in-depth insights into transcriptional pQTLs across multiple biological domains, shedding light on the genetic influence on ligand-receptor interactions and pathway disruptions involving cytokines and complement networks. The utility of these data in drug discovery was demonstrated through the extension of genetic proxy effects on protein targets like PCSK9 and the identification of gene and protein variations associated with susceptibility to diseases such as COVID-19.^[26]^

In this study, we examined 3 primary trait categories: circulating protein levels, COPD diagnosis, and lifestyle factors. For circulating proteins, sample sizes were as follows: Decode cohort: 35,559 individuals profiled across 4907 aptamers; UKB-PPP: 54,219 individuals with proteomic data on 2923 proteins; For COPD phenotype, summary statistics were derived from the FinnGen R11 release, comprising 2,69,077 participants, of whom 13,911 were COPD cases; For lifestyle factors, we utilized data from the IEU OpenGWAS project, with trait-specific sample sizes ranging from approximately 1,00,000 to 4,50,000 participants, depending on the exposure (see Table S1, Supplemental Digital Content, https://links.lww.com/MD/Q263 for exact n values). All summary-level GWAS data used in this study were derived from publicly available consortia and biobank resources. All data in this study are available in Supplementary Materials 1–8, Supplemental Digital Content, https://links.lww.com/MD/Q263. We confirm that no individual-level sample overlap exists between the Decode, UKB-PPP, and FinnGen datasets, as these represent independent cohorts. All GWAS summary statistics were adjusted for population structure using principal components of ancestry by their respective consortia. Additionally, to ensure population homogeneity and minimize stratification bias, we restricted our MR analysis to participants of European descent across all datasets.

### 2.3. Acquisition of cis- pQTLs

The data for cis-pQTLs used in this study were obtained directly from the original research studies as detailed in Section 2. These primary sources provide the foundational genetic associations between cis-pQTL and protein expression levels, ensuring accuracy and relevance in our downstream analyses.^[27]^

### 2.4. MR analysis

Genetic variants, or pQTLs, that show significant correlations with protein levels were identified through GWAS. These pQTLs are categorized into cis-pQTLs, which are located near the coding genes, and trans-acting protein quantitative trait loci, which are positioned farther from the genes. For MR analysis, cis-pQTLs were prioritized as instrumental variables due to their proximal association with gene expression. The TwoSampleMR package in R was employed to conduct the MR analysis between cis-pQTLs and COPD.^[28]^ For cis-pQTLs linked with only a single SNP, the Wald ratio method was applied as the primary analytical approach. In cases where multiple SNPs were available for a single cis-pQTL, the IVW method was utilized as the main approach, with additional MR methods applied as supplementary analyses to ensure robustness of the findings.^[29]^ Statistical significance was defined as *P* < .05, and all tests were 2-sided. To assess the strength of the instrumental variables used in MR, we calculated the *F*-statistics for each selected SNP. All instruments demonstrated strong relevance, with *F*-statistics exceeding the conventional threshold of 10 (mean *F* = 38.4; range: 21.7–74.2), indicating minimal risk of weak instrument bias. To correct for multiple testing in the proteome-wide MR analyses, we applied false discovery rate (FDR) adjustment using the Benjamini–Hochberg method. Associations with FDR-adjusted *P* values < .05 were considered statistically significant.

### 2.5. Co-localization analysis

To assess the co-localization of genetic variants within candidate gene regions across different phenotypes, such as disease susceptibility and gene expression levels, we performed a co-localization analysis. This analysis is designed to determine whether a shared genetic variant simultaneously influences 2 phenotypes, thereby suggesting a potential causal relationship.^[15]^ The coloc R package was used to conduct the co-localization analysis, calculating 5 posterior probabilities (PPs) for each region, each representing a different hypothesis: PP0: No association in the region; PP1: only the first trait is associated with the region, while the second trait is not; PP2: only the second trait is associated with the region, while the first trait is not; PP3: both traits are associated in the region but are influenced by different genetic variants; PP4: both traits are associated in the region and are influenced by the same genetic variant.^[16]^

In our analysis, we primarily focused on PPH3 and PPH4 values, as they provide insight into whether 2 traits are associated due to different or the same genetic variant, respectively. A high PPH4 value indicates strong evidence for a shared causal variant between the traits.^[30]^

### 2.6. Gene-based association and pathway analyses

To better understand the biological significance of the candidate target proteins, we utilized the GENE2FUNC tool within the FUMA web application interface. This tool allowed us to functionally annotate the candidate genes, providing insights into their roles in various biological processes and their degree of enrichment within specific pathways and tissues.^[31]^

In addition, we performed comprehensive pathway and tissue expression analyses to examine the functional contexts in which these proteins are active. For the enrichment analysis, we applied a FDR threshold of 0.05 to control for multiple testing. Furthermore, we set a requirement of at least 2 overlapping genes with the target gene set to confirm statistical significance, ensuring that the observed enrichment was meaningful and not due to random chance.^[32]^

### 2.7. Evaluation of medicinal properties of drugs and PPI

We began by using DrugBank to identify targets of commonly used drugs for treating COPD, focusing on proteins with strong evidence as potential therapeutic targets. Any protein that is currently targeted by approved drugs or investigational compounds was classified as a potential drug target for COPD.^[33]^ The relevant drug information for each identified protein was documented to facilitate further analysis.

Following this, we constructed a PPI network using the STRING database. This network enabled us to investigate whether the established drug targets interact with our newly identified candidate proteins, providing insights into potential pathways and interactions that could be leveraged for therapeutic purposes. By analyzing these interactions, we aimed to assess the feasibility of repurposing existing drugs or developing new therapeutics targeting these proteins to treat COPD more effectively.^[34]^

### 2.8. The impact of lifestyle on predicting protein targets

In addition, we performed MR analysis to explore the impact of various lifestyle factors on COPD-related proteins, aiming to identify proteins that could potentially be modulated through lifestyle interventions.^[35]^ A total of 17 lifestyle factors (listed in Supplementary Material 1, Supplemental Digital Content, https://links.lww.com/MD/Q263) were analyzed to evaluate their causal associations with target proteins implicated in COPD.

The methodology for this MR analysis followed the same approach as our broader proteome-wide MR analysis, ensuring consistency and robustness across all analyses. All statistical analyses were conducted using R software version 4.4.2, allowing us to systematically assess the influence of lifestyle factors on protein levels and identify potential targets for lifestyle-based intervention strategies in COPD management.^[36]^

## 3. Result

### 3.1. Mendelian randomization analysis of plasma proteins and COPD

**Figure 2.**
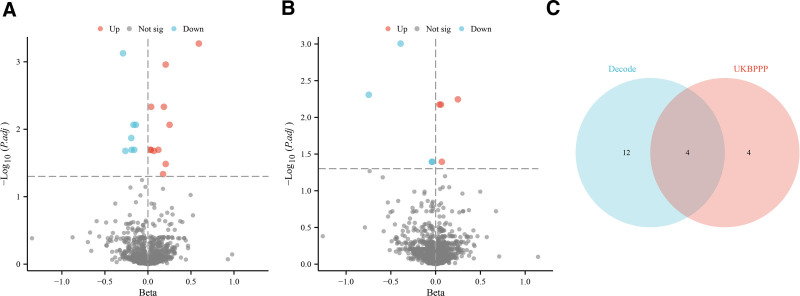
Positive causal relationships between circulating proteins and COPD after FDR correction. (A) Volcano plot showing significant protein associations with COPD identified in the Decode project after FDR correction, with upregulated proteins in red and downregulated proteins in blue. (B) Volcano plot for significant associations from the UKB-PPP cohort, following FDR correction, displaying the direction and magnitude of effects. (C) Venn diagram indicating the overlap of proteins associated with COPD across Decode and UKB-PPP datasets, showing 4 shared protein markers, emphasizing consistency in results across cohorts. COPD = chronic obstructive pulmonary disease, FDR = false discovery rate, UKB-PPP = UK Biobank Pharmaceutical Proteomics Project.

**Figure 3.**
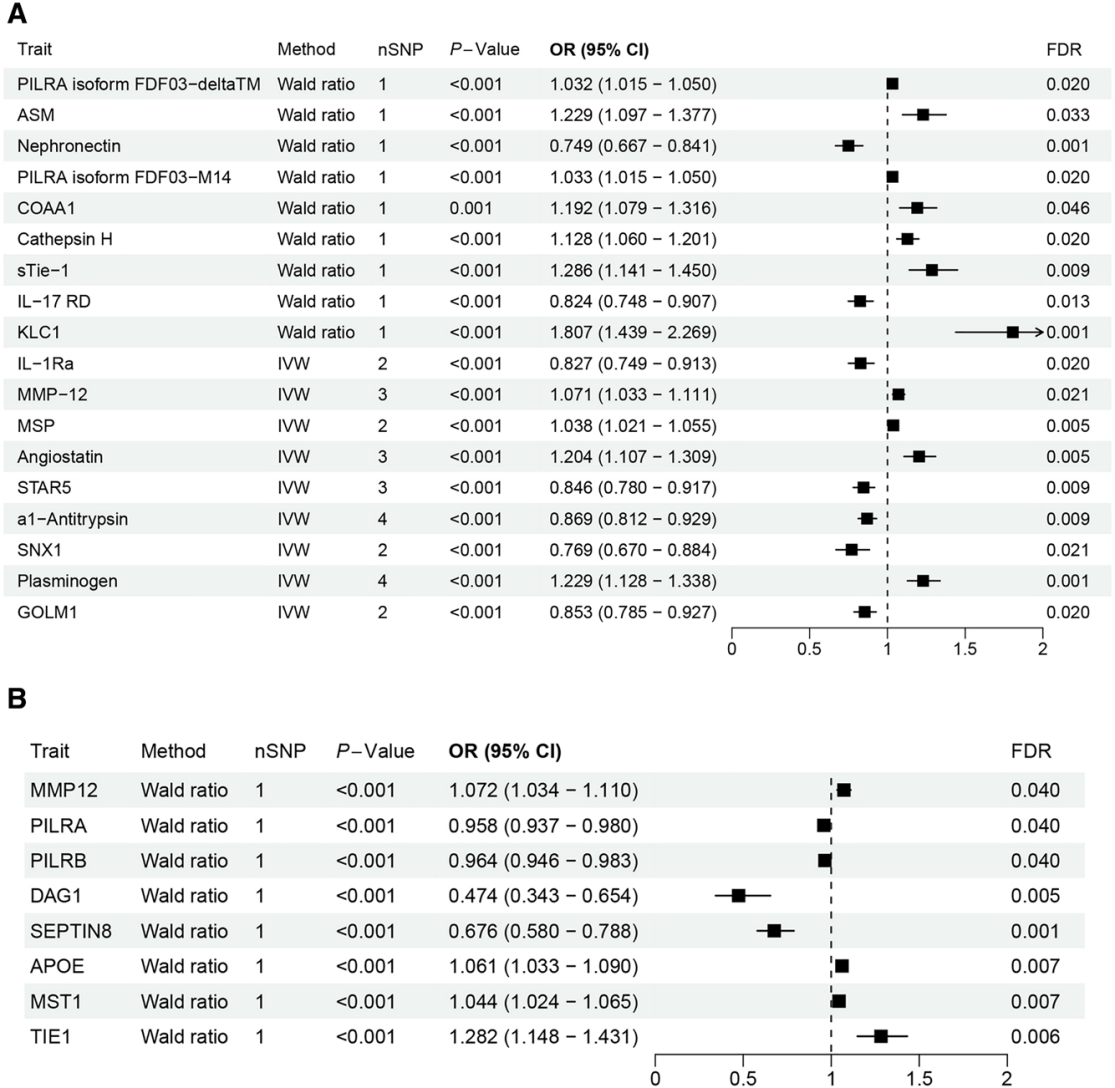
Analysis of causal relationships between circulating proteins and COPD after FDR correction. (A) Forest plot depicting odds ratios and confidence intervals (CIs) for proteins from the Decode dataset with significant associations to COPD. (B) Forest plot for the UKB-PPP dataset, illustrating significant protein associations with COPD, supporting the Decode findings and reinforcing the validity of these proteins as potential COPD biomarkers. CI = confidence interval, COPD = chronic obstructive pulmonary disease, FDR = false discovery rate, UKB-PPP = UK Biobank Pharmaceutical Proteomics Project.

In the Decode project’s analysis of cis-pQTL associations with COPD, we initially identified associations between 155 plasma proteins and COPD (*P* < .05, see Supplementary Material 2, Supplemental Digital Content, https://links.lww.com/MD/Q263). Following adjustment for the FDR, 18 proteins maintained statistically significant associations with COPD (FDR < 0.05; illustrated in Figs. 2A and 3A). To further explore protein associations with COPD, we utilized the trans-acting protein quantitative trait loci data from the UKB-PPP cohort, which initially revealed associations between 169 proteins and COPD (*P* < .05, Supplementary Material 3, Supplemental Digital Content, https://links.lww.com/MD/Q263). After FDR correction, 8 proteins retained significant associations with COPD (FDR < 0.05; depicted in Figs. 2B and 3B). Notably, an overlap of 4 proteins was observed between the Decode and UKB-PPP datasets, indicating robust associations across both cohorts (Fig. 2C). The final causal relationships between these proteins and COPD, adjusted for Decode and UKB-PPP data, are presented in Figure 3.

A Steiger filtering test was subsequently conducted to verify the causal direction, confirming the absence of reverse causality in the findings from both Decode (Supplementary Material 4, Supplemental Digital Content, https://links.lww.com/MD/Q263) and UKB-PPP (Supplementary Material 5, Supplemental Digital Content, https://links.lww.com/MD/Q263) datasets. This stringent analysis enhances the reliability of our identified protein biomarkers associated with COPD, which are now poised for further functional and therapeutic exploration.

### 3.2. Co-localization results

**Figure 4.**
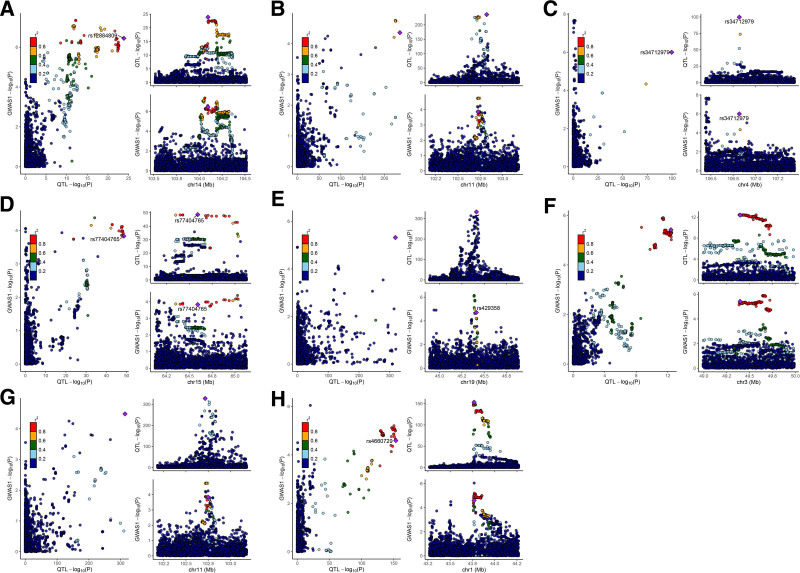
Co-localization analysis of circulating proteins and COPD with strong evidence of causal association (PPH4 > 0.7). (A) KLC1 in the Decode dataset (PPH4 = 0.848), demonstrating a significant association with COPD. (B) MMP12 in the Decode dataset (PPH4 = 0.802), showing strong co-localization with COPD susceptibility loci. (C) Nephronectin (NPNT) in the Decode dataset (PPH4 = 0.744), indicating a potential causal role in COPD. (D) SNX1 in the Decode dataset (PPH4 = 0.908), with high co-localization probability for COPD. (E) APOE in the UKB-PPP dataset (PPH4 = 0.885), supporting its involvement in COPD. (F) DAG1 in the UKB-PPP dataset (PPH4 = 0.888), providing further evidence of COPD association. (G) MMP12 in the UKB-PPP dataset (PPH4 = 0.885), reinforcing its causal link to COPD. (H) TIE1 in the UKB-PPP dataset (PPH4 = 0.892), highlighting its potential relevance to COPD pathology. APOE = apolipoprotein E, COPD = chronic obstructive pulmonary disease, MMP12 = matrix metalloproteinase-12, NPNT = NPNT = nephronectin, UKB-PPP = UK Biobank Pharmaceutical Proteomics Project.

Based on the co-localization analysis of proteins that remained significantly associated with COPD after FDR correction, we defined PPH4 > 0.7 as strong evidence of a causal relationship. Within the Decode plasma protein dataset, we identified 4 proteins with strong evidence of a causal link to COPD: KLC1 (PPH4 = 0.848, Fig. 4A), MMP12 (PPH4 = 0.802, Fig. 4B), nephronectin (NPNT, PPH4 = 0.744, Fig. 4C), and SNX1 (PPH4 = 0.908, Fig. 4D), as detailed in Supplementary Material 6, Supplemental Digital Content, https://links.lww.com/MD/Q263.

In the UKB-PPP plasma protein data, 5 proteins demonstrated common causal variations with COPD, further reinforcing the association: APOE (PPH4 = 0.885, Fig. 4E), DAG1 (PPH4 = 0.888, Fig. 4F), MMP12 (PPH4 = 0.885, Fig. 4G), and TIE1 (PPH4 = 0.892, Fig. 4H), with additional details provided in Supplementary Material 7, Supplemental Digital Content, https://links.lww.com/MD/Q263. This co-localization evidence supports a potential causal role of these proteins in COPD, warranting further investigation into their mechanisms and therapeutic relevance.

### 3.3. Gene-based association and pathway analyses

**Figure 5.**
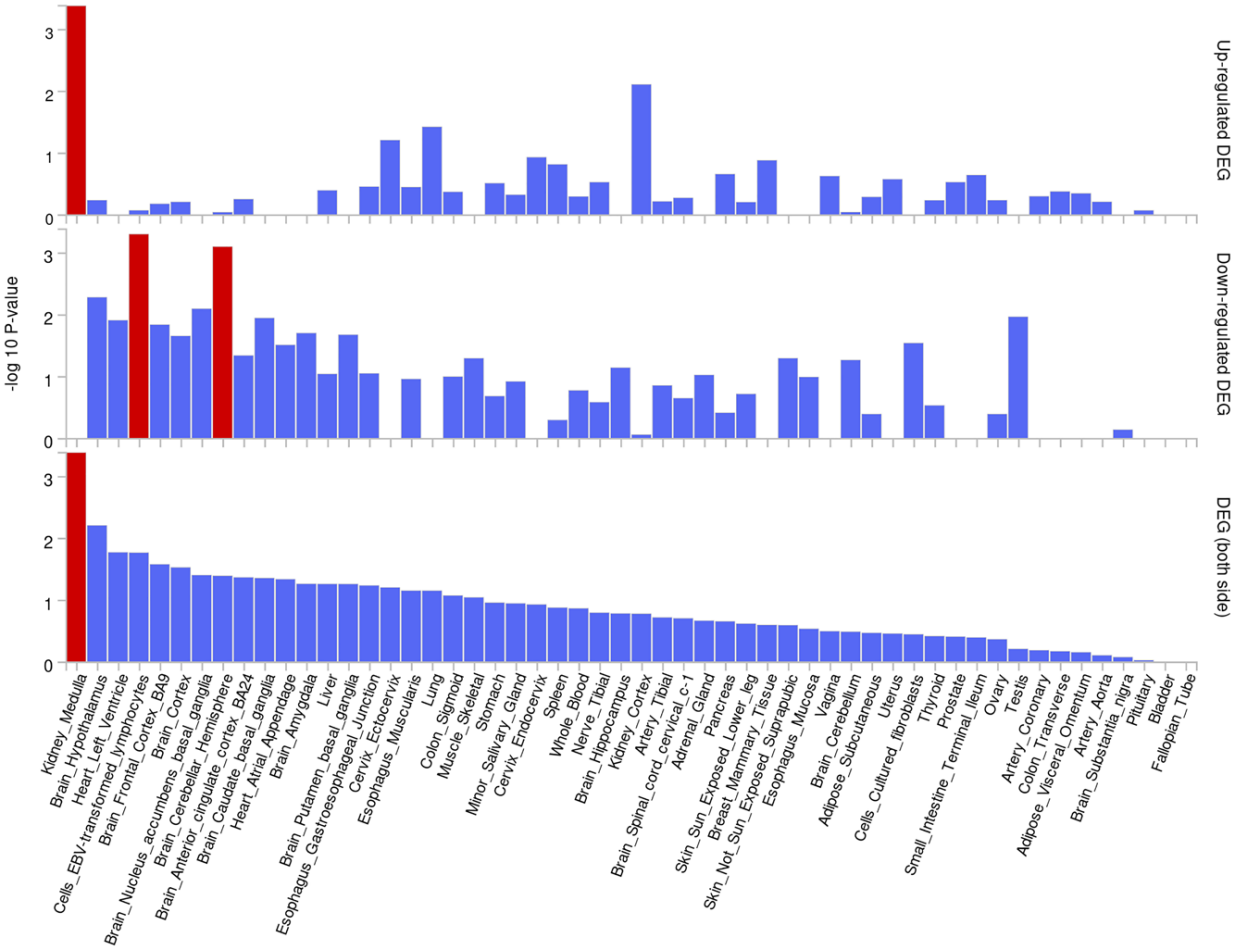
Differential expression analysis of key genome-wide significant loci and protein-coding genes identified in Decode and UKB-PPP. This figure displays the expression profiles of significant genes across 54 GTEx v8 tissues. Red bars indicate tissues where the gene set exhibits differential expression, potentially highlighting tissue-specific relevance in COPD pathology. COPD = chronic obstructive pulmonary disease, UKB-PPP = UK Biobank Pharmaceutical Proteomics Project.

**Figure 6.**
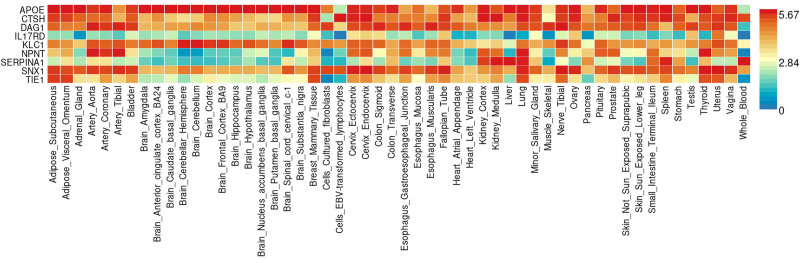
Heatmap of gene expression for significant proteins identified in Decode and UKB-PPP datasets. This figure provides a comparative view of expression levels across various tissues, illustrating the differential expression patterns of genes such as APOE, CTSH, DAG1, IL17RD, KLC1, NPNT, SERPINA1, SNX1, and TIE1, which may be critical in lung-related health and COPD progression. APOE = apolipoprotein E, COPD = chronic obstructive pulmonary disease, NPNT = nephronectin, UKB-PPP = UK Biobank Pharmaceutical Proteomics Project.

Based on the selection criteria of PPH3 + PPH4 > 0.9 to ensure strong evidence of causality, our analysis identified genes with significant differential expression across various tissues. Figures 5 and 6 illustrate this tissue-specific expression, highlighting the relevance of these genes within multiple organs and biological contexts. As shown in Figure 6, the heatmap provides a comparative view of expression levels across tissues, with genes such as apolipoprotein E (APOE), CTSH, DAG1, IL17RD, KLC1, NPNT, SERPINA1, SNX1, and TIE1 exhibiting notable variance.

**Figure 7.**
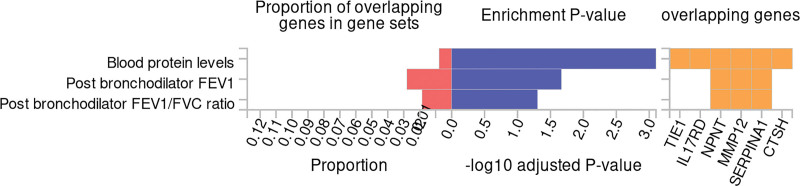
Pathway enrichment analysis for unique proteins identified in Decode and UKB-PPP. This figure emphasizes the involvement of these genes in lung function-associated pathways, including those linked to respiratory mechanics, immune response, and inflammation regulation, underscoring their potential as therapeutic targets in COPD and other respiratory diseases. COPD = chronic obstructive pulmonary disease, UKB-PPP = UK Biobank Pharmaceutical Proteomics Project.

Furthermore, the GWAS catalog data suggests that these genes are highly enriched in pathways associated with lung function, reinforcing their potential role in respiratory health and COPD pathology. Figure 7 underscores this enrichment, particularly emphasizing pathways related to respiratory mechanics, immune response, and inflammatory regulation. This functional annotation and pathway analysis thus provide insight into the biological roles these genes may play, offering potential therapeutic targets within lung-related diseases.

### 3.4. Drug targets and PPI network construction

**Figure 8.**
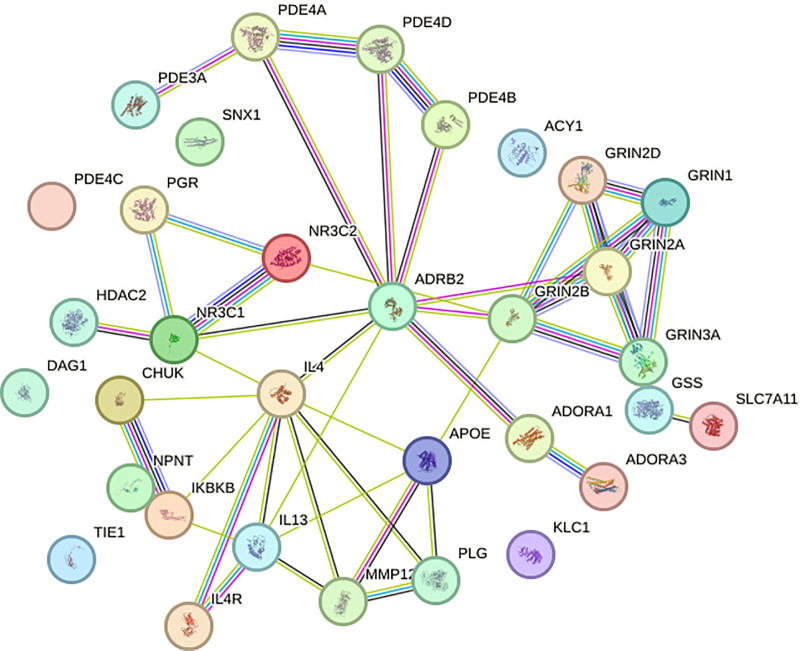
PPI network analysis of existing drug targets and newly predicted protein targets. This network illustrates the interactions between selected drug targets and COPD-associated proteins identified through our analysis. Notable connections include the *N*-acetylcysteine target GRIN2B and the dupilumab targets IL13 and IL4, which exhibit direct interactions with predicted protein targets, suggesting their potential as therapeutic intervention points. In contrast, targets for other drugs displayed minimal to no direct interactions with identified candidate proteins, underscoring the specificity of certain proteins, such as GRIN2B, IL13, and IL4, in COPD-related pathways. COPD = chronic obstructive pulmonary disease, PPI = protein–protein interaction.

Based on the PPI network analysis depicted in Figure 8 (Supplementary Material 8, Supplemental Digital Content, https://links.lww.com/MD/Q263), we observed interactions among specific drug targets and the predicted candidate proteins. Notably, the *N*-acetylcysteine target GRIN2B and the dupilumab targets IL13 and IL4 demonstrated direct connections within the network. However, analysis revealed that the remaining targets for the other drugs displayed no significant interactions with the identified candidate proteins. This suggests that while some therapeutic interactions may be directly influenced by these protein targets, others lack a direct connection, emphasizing the potential specificity of certain proteins, such as GRIN2B, IL13, and IL4, as therapeutic targets for COPD-related interventions.

### 3.5. The impact of lifestyle on 3 types of proteins

**Figure 9.**
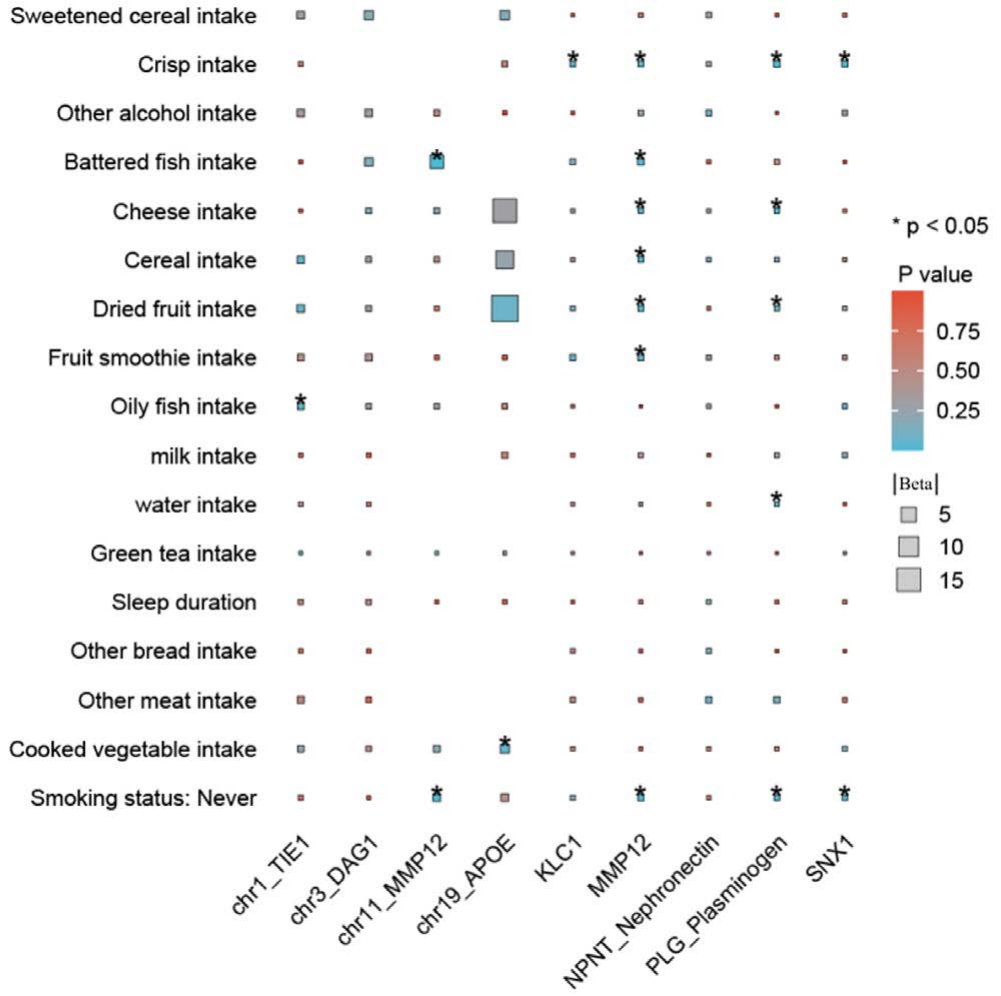
The impact of lifestyle factors on predicted COPD-related protein targets. This figure presents the Mendelian randomization analysis examining causal relationships between various lifestyle factors and specific COPD-related protein loci. For the UKB-PPP dataset, the analysis was restricted to chromosome-classified data, focusing on chromosomes with relevant protein genes. In the Decode dataset, a comprehensive analysis was conducted across all data. Significant findings include causal associations between oily fish intake and TIE1 on chromosome 1; battered fish intake and never smoking with MMP12 on chromosome 11; and cooked vegetable intake with APOE on chromosome 19. Additionally, crisp intake was linked to multiple proteins, including KLC1, MMP12, NPNT, PLG, and SNX1. Other associations were observed between cheese intake, dried fruit intake, water intake, and proteins such as MMP12, NPNT, and PLG. These results highlight the potential of dietary and lifestyle interventions in modulating COPD-related proteins, providing insights into managing COPD risk factors. APOE = apolipoprotein E, COPD = chronic obstructive pulmonary disease, MMP12 = matrix metalloproteinase-12, NPNT = nephronectin, UKB-PPP = UK Biobank Pharmaceutical Proteomics Project.

Based on the data presented in Figure 9, we extended our MR analysis to explore causal relationships between lifestyle factors and specific COPD-related loci protein. For the UKB-PPP dataset, restricted to chromosome-classified data, we focused on the chromosomes where relevant protein genes are located. In contrast, we examined all available Decode data.

The analysis identified several significant associations: oily fish intake showed a causal relationship with TIE1 on chromosome 1; battered fish intake and never smoking were associated with MMP12 on chromosome 11; cooked vegetable intake demonstrated a causal association with APOE on chromosome 19; crisp intake exhibited causal links with KLC1 as well as multiple proteins, including MMP12, NPNT, PLG (plasminogen), and SNX1; additional associations included cheese intake, dried fruit intake, and water intake, all demonstrating causal relationships with MMP12, NPNT, and PLG.

This data underscores the potential influence of specific lifestyle factors, such as dietary habits and smoking status, on COPD-related proteins, suggesting avenues for targeted lifestyle interventions in managing COPD risks and progression.

## 4. Discussion

This comprehensive investigation provides compelling evidence for the causal involvement of specific circulating proteins in the pathogenesis of COPD. By integrating large-scale genetic data from the Decode and UKB-PPP cohorts, the study identified a robust set of plasma proteins that exhibit causal relationships with COPD susceptibility. The application of MR analysis, combined with rigorous co-localization assessment, strengthens the validity of these findings and underscores the potential of these proteins as novel biomarkers and therapeutic targets for COPD.

A key strength of this study is the utilization of 2 independent datasets, which enabled the researchers to replicate and validate the causal associations between circulating proteins and COPD.^[37]^ The identification of 18 proteins from the Decode dataset and 8 proteins from the UKB-PPP cohort that maintained significant associations with COPD after FDR correction highlights the consistency and robustness of these findings.^[38]^ Notably, the overlap of 4 proteins between the 2 datasets, including MMP12, further reinforces the potential importance of these proteins in COPD pathogenesis.^[39]^

The co-localization analysis provided an additional layer of evidence supporting the causal relationships between specific proteins and COPD.^[40]^ By demonstrating strong evidence of shared genetic variants influencing both protein levels and COPD susceptibility, the co-localization findings lend credence to the proposed causal mechanisms.^[41]^ Proteins such as KLC1, MMP12, NPNT, SNX1, APOE, DAG1, and TIE1 emerged as having high PPs of co-localization (PPH4 > 0.7), suggesting that they are likely to play direct causal roles in COPD development.^[42]^

The functional characterization of the identified proteins through tissue-specific differential expression and pathway analyses yielded valuable insights into their potential biological relevance in COPD. The observed enrichment of these proteins in pathways related to respiratory mechanics, immune response, and inflammatory regulation aligns with the known pathological hallmarks of COPD, including airflow limitation, chronic inflammation, and tissue remodeling.^[43]^ This functional annotation highlights the mechanistic underpinnings by which these proteins may contribute to the complex pathogenesis of COPD.^[44]^

For instance, the elevated expression of MMP12 (matrix metalloproteinase-12) in lung tissue is consistent with its well-established role in COPD.^[44]^ MMP12 is known to mediate extracellular matrix degradation, leading to alveolar destruction and emphysema, a key feature of COPD. In addition to its well-established role in extracellular matrix degradation, MMP12 may also act as a mediator of oxidative stress – a key pathological feature of COPD. Oxidative stress results from an imbalance between reactive oxygen species production and antioxidant defenses, contributing to airway inflammation, epithelial cell injury, and structural remodeling. Notably, proteomic findings suggest that MMP12 may participate in oxidative pathways, supporting its dual function in both matrix degradation and redox imbalance. Previous studies reported elevated levels of oxidative stress markers like 8-isoprostane and hydrogen peroxide (H₂O₂) in exhaled breath condensate from both smokers and COPD patients, emphasizing the continuum of oxidative damage even prior to spirometric impairment.^[45]^ These findings suggest that MMP12 may link inflammatory and oxidative responses in the early stages of COPD development, thereby reinforcing its potential as a multidimensional therapeutic target. Additionally, the involvement of APOE in lipid metabolism and inflammation pathways suggests its potential influence on COPD-related comorbidities, such as cardiovascular disease.^[46]^ The differential expression patterns of these proteins across various tissues, including the lung, highlight their multifaceted roles in respiratory health and disease.^[47]^ Despite the robust findings derived from large-scale cohorts, we acknowledge that the generalizability of our results is currently limited to individuals of European ancestry. This is due to the ancestry composition of the Decode, UKB-PPP, and FinnGen datasets. Future studies are warranted to validate our findings in diverse ancestral groups, such as East Asian or African populations, using resources like Biobank Japan, the GERA cohort, or the PAGE study. Such efforts will be essential to assess the broader applicability of these protein biomarkers and enhance the translational relevance of our results across global populations.

Furthermore, the exploration of potential drug targets and PPIs provided valuable insights into the therapeutic relevance of the identified proteins. The finding that the *N*-acetylcysteine target GRIN2B and the dupilumab targets IL13 and IL4 exhibited direct connections within the PPI network suggests that modulating these specific proteins may be a promising approach for COPD management.^[48]^ In contrast, the lack of significant interactions between the remaining drug targets and the candidate proteins emphasizes the potential for identifying novel therapeutic targets among the newly discovered COPD-associated proteins.^[49]^

Importantly, the systematic MR analysis examining the impact of lifestyle factors on COPD-related proteins offers unique and clinically relevant insights. The identification of causal associations between specific dietary factors, such as oily fish intake, battered fish intake, cooked vegetable intake, and crisp intake, with proteins like TIE1, MMP12, APOE, KLC1, NPNT, PLG, and SNX1, underscores the potential for lifestyle-based interventions to modulate key proteins involved in COPD pathogenesis.^[50]^

These findings suggest that targeted dietary modifications may represent a promising avenue for COPD management, potentially complementing pharmacological therapies.^[51]^ By understanding the specific proteins that can be influenced by lifestyle factors, healthcare providers can devise personalized, multifaceted treatment strategies that empower patients to take an active role in managing their disease through lifestyle changes.^[52]^ This knowledge could inform the development of novel, integrated care models that seamlessly combine pharmaceutical interventions with tailored lifestyle recommendations.^[53]^

The comprehensive nature of this study, encompassing both genetic epidemiology and functional analyses, provides a robust foundation for future research and translational applications.^[54]^ The identified causal protein biomarkers hold promise for enhancing early COPD detection and risk stratification, enabling timely intervention and personalized management strategies.^[55]^ Furthermore, the elucidation of the mechanistic roles of these proteins in COPD pathogenesis may guide the development of novel targeted therapies, potentially leading to more effective treatments for this debilitating respiratory condition.^[56]^

One of the limitations of this study is the reliance on existing genetic and proteomic datasets, which may not fully capture the complexity of COPD pathogenesis. Additional longitudinal studies and validation in diverse patient populations would further strengthen the generalizability of the findings.^[57]^ Moreover, the identification of causal relationships does not necessarily imply that modulating the levels of these proteins will lead to improved clinical outcomes; experimental studies and therapeutic development efforts are still required to establish the efficacy of targeting these proteins for COPD management.^[58]^

Nevertheless, this comprehensive investigation represents a significant step forward in the understanding of COPD pathogenesis and the identification of novel therapeutic avenues.^[59]^ By integrating cutting edge genetic and bioinformatic approaches, the researchers have shed light on the causal roles of circulating proteins in COPD susceptibility and uncovered potential lifestyle-based intervention strategies.^[60]^ These findings have important implications for the development of improved COPD screening tools, targeted pharmacological treatments, and personalized, multifaceted management approaches that leverage both pharmaceutical and lifestyle-based interventions.^[61]^

In conclusion, this study has made a substantial contribution to the field of COPD research by elucidating the causal relationships between circulating proteins and disease susceptibility, as well as exploring the modulating influence of lifestyle factors. The identified protein biomarkers and their associated biological pathways provide a valuable foundation for future investigations aimed at translating these findings into enhanced COPD prevention, diagnosis, and treatment strategies. By bridging the gap between genetic epidemiology and functional biology, this research paves the way for a more comprehensive understanding of COPD pathogenesis and the development of innovative, personalized approaches to improve patient outcomes and reduce the global burden of this debilitating respiratory condition.

## Author contributions

**Conceptualization:** Qingxiu Zheng, Wenqi Ouyang, Binbin Chen.

**Data curation:** Qingxiu Zheng, Jiangpo Ma, Wenqi Ouyang, Binbin Chen.

**Formal analysis:** Jiangpo Ma.

**Methodology:** Jiangpo Ma.

**Validation:** Qingxiu Zheng, Wenqi Ouyang.

**Visualization:** Xuejiao Lin.

**Writing – original draft:** Xuejiao Lin, Binbin Chen.

**Writing – review & editing:** Xuejiao Lin, Binbin Chen.

## Supplementary Material


